# Analysis of the immune microenvironment in resected non-small cell lung cancer: the prognostic value of different T lymphocyte markers

**DOI:** 10.18632/oncotarget.10811

**Published:** 2016-07-24

**Authors:** Marta Usó, Eloisa Jantus-Lewintre, Roy M. Bremnes, Silvia Calabuig, Ana Blasco, Enrique Pastor, Irene Borreda, Sonia Molina-Pinelo, Luis Paz-Ares, Ricardo Guijarro, Miguel Martorell, Jerónimo Forteza, Carlos Camps, Rafael Sirera

**Affiliations:** ^1^ Department of Medicine, Universitat de València, Valencia, Spain; ^2^ Molecular Oncology Laboratory, Fundación Investigación, Hospital General Universitario de Valencia, Valencia, Spain; ^3^ Department of Biotechnology, Universitat Politècnica de València, Valencia, Spain; ^4^ Department of Oncology, University Hospital of North Norway, Tromso, Norway; ^5^ Department of Clinical Medicine, The Arctic University of Norway, Tromso, Norway; ^6^ Department of Pathology, Universitat de València, Valencia, Spain; ^7^ Medical Oncology Department, Hospital General Universitario de Valencia, Valencia, Spain; ^8^ Department of Thoracic Surgery, Hospital General Universitario de Valencia, Valencia, Spain; ^9^ Instituto Valenciano de Patología, Universidad Católica de Valencia, Unidad Mixta de Patología Molecular Centro de Investigación Príncipe Felipe (CIPF)-Universidad Católica de Valencia (UCV), Valencia, Spain; ^10^ Medical Oncology Department, Hospital 12 de Octubre & Centro Nacional de Investigaciones Oncológicas (CNIO), Madrid, Spain; ^11^ Universidad Complutense de Madrid, Madrid, Spain; ^12^ Department of Pathology, Hospital General Universitario de Valencia, Valencia, Spain

**Keywords:** NSCLC, prognostic, immune-biomarker, tumor stroma, tumor compartment

## Abstract

The prognosis of non-small cell lung cancer (NSCLC) remains poor and heterogeneous and new biomarkers are needed. As the immune system plays a pivotal role in cancer, the study of immune-related markers may provide valuable prognostic information of NSCLC. In 122 formalin-fixed, paraffin-embedded tumor tissue samples from early-stage NSCLC, tumor and tumor-near stromal areas were microdissected and gene expression levels of conventional and regulatory T cell markers were assessed by quantitative polymerase chain reaction. Also, the presence of infiltrating CD4+, CD8+, and FOXP3+ cells in tumor samples was assessed by immunohistochemistry. The relative proportion of conventional and regulatory T cells present in the tumor environment was assessed and found to be key to understand the importance that the immune system analysis has in the prognostics of NSCLC patients. The presence of CD8+ cells in the tumor compartment was associated with better outcome, whereas the presence of FOXP3+ cells was associated with worse overall survival. The negative prognostic value of combined biomarkers, indicating high levels of FOXP3 in the stroma and low levels of CD4 or CD8 in tumors, was observed at mRNA level and was validated by immunohistochemistry.In conclusion, the proportion of T helper and cytotoxic cells vs. regulatory T cells in different locations of the tumor microenvironment have opposite prognostic impacts in resected NSCLC.

## INTRODUCTION

Lung cancer is the leading cause of cancer-related death worldwide [1]. Five-year survival remains poor, mainly due to late debut of symptoms and the presence of regional or distant metastases at diagnosis [2]. An increasing research effort has been invested in order to detect and validate novel biomarkers in non-small cell lung cancer (NSCLC). Although some of these markers have been adopted in current clinical practice, several potential biomarkers have not yet been adequately validated [3, 4]. Both tumor-preventing and tumor-promoting inflammation has been proved to play prognostic roles in several solid tumors [5-8]. Tumor-infiltrating cytotoxic T cells are of crucial importance in suppressing cancer development and controlling disease progression. Among the different cell subsets of tumor infiltrating lymphocytes (TILs), the most promising biomarker so far are CD8+ cytotoxic T lymphocytes, that are a pivotal component of the cell-mediated antitumor immune responses [9]. In NSCLC, together with other malignancies, many studies have evaluated the clinical value of CD8+ cells alone or combined with other biomarkers, demonstrating a strong independent positive prognostic effect regardless of tissue compartments evaluated [10-16].

On the other hand, regulatory T cells (Tregs) are a subtype of CD4+T cells with immunosuppressive properties that express high levels of CD25 and low levels of CD127 on their cell surface [17, 18]. One of the most reliable phenotypic markers for Tregs is FOXP3, which is essential for their development and function and which has been broadly used for their detection [19]. Tregs are also characterized by the expression of other markers such as cytotoxic T-lymphocyte-associated antigen 4 (CTLA4), lymphocyte activation gene 3 (LAG3) and glucocorticoid induced tumor necrosis factor receptor family related gene (GITR), or soluble immunosuppressive factors such as interleukin 10 (IL10) and transforming growth factor beta 1 (TGBF1) [20]. The presence of FOXP3+ lymphocytes in cohorts of patients affected by different types of tumors, has been associated with a poor prognosis [21-25] or a better locoregional control and prognosis [26-29].

Altogether, the data obtained so far indicates that immune system-related biomarkers, and more specifically those involved in immunoregulation, may become useful in both development of the next generation biomarkers in NSCLC and as potential targets for novel therapies. Furthermore, it should be taken into consideration that the prognostic and predictive value of a given biomarker may be different or even opposite depending on which cellular compartment of the tumor it is expressed. Therefore, further studies using feasible and reliable techniques such as quantitative real-time PCR (RTqPCR), based on microdissected specimen, or immunohistochemistry (IHC) are essential to detect and validate new biomarkers. The aim of this study was to analyze the presence of immune markers at the mRNA and protein level in the tumor and tumor-near stroma compartment separately, and to investigate the relative proportion of conventional and regulatory T cells present in the tumor surrounding in order to understand their associations with the patients’ prognosis in an unselected cohort of resected NSCLC patients.

## RESULTS

### Patient characteristics

Detailed clinical and pathological information including age, gender, stage of disease and histology are summarized in Table 1. The median follow-up was 53.3 [1-113] months. Of the 122 resected NSCLC patients included, 68 (55.7%) relapsed and 67 (54.9%) died during the follow-up period.

**Table:** Clinicopathological characteristics of the patients

Characteristics	*N*	%
Age at surgery (median, range)	65 [26-85]	
**Gender**		
Male	104	85.4
Female	18	14.6
**Stage**		
I	72	59
II	26	21.3
IIIA	24	19.7
**Histology**		
SCC	58	47.5
ADC	51	41.8
Others	13	10.7
**Performance Status**		
0	70	57.4
1-2	35	28.7
NS	17	13.9
**Differentiation grade**		
Poor	29	23.8
Moderate	48	39.3
Well	28	23
NS	17	13.9
**Smoking Status**		
Current	59	48.4
Former	46	37.7
Never-smoker	17	13.9
**EGFR**		
Wild type	43	35.2
Mutated	9	7.4
NA	70	57.4
**KRAS**		
Wild type	83	68
Mutated	14	11.5
NA	25	20.5

ADC, adenocarcinoma; SCC, squamous; NA; not analyzed; NS, non-specified; EGFR, epidermal growth factor receptor.

### Relative gene expression

All of the analyzed genes could be amplified using the selected primers/probes. We observed a strong overexpression, in both the tumor-near stroma and the tumor (measured as fold-change, X and range) of CD25 (19.46X, [2.7-86.0], 11.59X, [0.3-54.2]), FOXP3 (4.96X, [0.2-67.4], 4.08X, [0.1-38.0]), CTLA4 (2.89X, [0.2-65.7], 3.02X, [0.1-59.8]) and TGFB1 (3.24X, [0.6-16-6], 2.117X, [0.2-27.2]). In contrast, IL10 expression in the tumor compartment was downregulated (0.39X, [0.02-40.3]).

### CD4+, CD8+, and FOXP3+ cell infiltration

**Figure 1:**
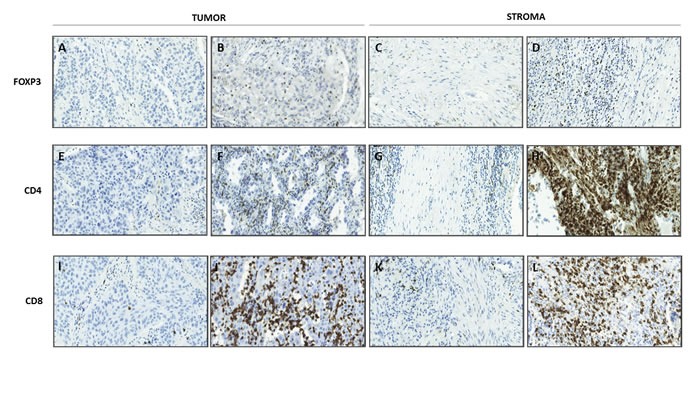
Representative immunohistochemical staining of FOXP3, CD4 and CD8 in tumor and stroma compartments Original magnification X200. **A.** Low infiltration of FOXP3^+^ lymphocytes in tumor compartment, **B.** high infiltration of FOXP3^+^ lymphocytes in tumor compartment, **C.** low infiltration of FOXP3^+^ lymphocytes in stroma compartment, **D.** high infiltration of FOXP3^+^ lymphocytes in stroma compartment, **E.** low infiltration of CD4^+^ lymphocytes in tumor compartment, **F.** high infiltration of CD4^+^ lymphocytes in tumor compartment, **G.** low infiltration of CD4^+^ lymphocytes in stroma compartment and, **H.** high infiltration of CD4^+^ lymphocytes in stroma compartment, **I.** Low infiltration of CD8^+^ lymphocytes in tumor compartment, **J.** high infiltration of CD8^+^ lymphocytes in tumor compartment, **K.** low infiltration of CD8^+^ lymphocytes in stroma compartment, **L.** high infiltration of CD8^+^ lymphocytes in stroma compartment.

In tonsil tissue samples, positive staining for CD4 and CD8 was observed in the cytoplasm of lymphocytes, whereas FOXP3 staining was observed in the nucleus; the same pattern was seen in patient samples. There was no positive staining in the negative controls. Figure 1 shows a representative immunohistochemical staining of FOXP3, CD4 and CD8 in tumor and stroma compartments. CD4+ and CD8+ cells were detected in all assessable samples, although in 13 (15.5%) of the 84 samples CD4+ cells were only found in the tumor-near stromal compartment. FOXP3+ cells were detected in 80 (95.2%) of the 84 assessable samples, although in 8 (10%) positive cells were only found in the tumor-near stromal compartment, and in one case (1.8%) positive cells were only found in the tumor compartment. For the three markers, there were significantly more positive T cells in the tumor-near stroma than in the tumor compartment (pair-wise Wilcoxon test, *p* < 0.001). The number of positive cells per HPF in the stromal compartment ranged from 1 to 76 (median: 18.8) for CD4, from 3 to 73 (median: 29.8, mean: 29) for CD8 and from 0 to 45 (median: 11.6) for FOXP3. On the other hand, in the tumor compartment the number ranged from 0 to 21 (median: 1.8, mean: 3.5) for CD4, from 1 to 82 (median: 5.6) for CD8 and from 0 to 15 (median: 1, mean: 1.6) for FOXP3.

Patients’ tumors were classified as being weakly to strongly infiltrated by CD4+ and CD8+ immune cells in tumor and tumor-near stroma compartments according to the median calculated for each marker. For FOXP3 expression in the stromal compartment, 6% of the samples were negative, 55% expressed in less than 10% of lymphocytes, 38% between 10% and 33%, and 1.2% in more than 33%. For the tumor compartment, 14% were negative, 71% expressed in less than 10% positive lymphocytes, 13% in 10% and 33%, and 1.2% in more than 33%.

### Correlation with clinicopathological variables

The expression, in both tumor and stromal compartments, of CD4 (*p* = 0.024 and *p* = 0.008, respectively), CD8 (*p* = 0.044 and *p* = 0.008, respectively), and LAG3 (*p* = 0.008, in both cases) were higher in adenocarcinoma (ADC) than in squamous cells carcinoma (SCC) patients. Moreover, higher IL10 expression was found in patients with stage I than stage II/IIIA disease (*p* = 0.027). As for the correlations with positive cell infiltration, we observed that higher levels (above the median) of CD4+ cells in the tumor stroma correlated with epidermal growth factor receptor (EGFR)-mutated patients (*p* = 0.047) and with ADC histology (*p* = 0.030). Furthermore, smaller tumors (less than 3.5 cm) were associated with a higher number of stromal CD8+ cells (*p* = 0.047).

### Prognostic value of gene expression markers and immune cell infiltration in tumor and tumor-near stroma

Survival analyses indicated that patients with high CD4 expression in the tumor compartment, dichotomized according to the median of its relative expression, had improved PFS (37.8 *vs*. 23 months, *p* = 0.042) and OS (81.2 *vs*. 42.9 months, *p* = 0.018). Similarly, high levels of CD8 expression in the tumor compartment were associated with improved PFS (81.2 *vs*. 19.4 months, *p* = 0.001) and OS (81.2 *vs*. 37.2 months, *p* < 0.001). The expression of CD8 in the stroma was also correlated with OS (74.3 *vs*. 46.4 months, *p* = 0.032). With respect to FOXP3 high expression levels were correlated with longer PFS (35.3 *vs*. 22.1 months, *p* = 0.020) and OS (NR *vs*. 37.2 months, *p* = 0.005).. Moreover, patients with high expression levels of LAG3 presented a better OS (69 *vs*. 36.2 months, *p* = 0.023), and the same was observed for TGFB1 (74.3 *vs*. 46.6 months, *p* = 0.032; Table 2). Kaplan-Meier plots for all these biomarkers are shown in supplementary Figure 1.

**Table 2:** Univariate analysis of the gene expression and immune cells infiltration-related biomarkers

	OS		PFS	
Variable	Median survival (months)	*p*-value	Median survival (months)	*p*-value
**Gene expression markers (*N*=122)**				
Tumoral CD4 High vs. low	81.2 vs. 42.9	0.018	37.8 vs. 23	0.042
Tumoral CD8 High vs. low	81.2 vs. 37.2	<0.001	81.2 vs. 19.4	0.001
Stromal CD8 High vs. low	74.3 vs. 46.4	0.032	29.1 vs. 25.6	0.237
Tumoral FOXP3 High vs. low	NR vs. 37.2	0.005	35.3 vs. 22.1	0.020
Tumoral LAG3 High vs. low	69 vs. 36.2	0.023	30.1 vs. 22.1	0.107
Tumoral TGBF1 High vs. low	74.3 vs. 46.6	0.032	31.5 vs. 22.1	0.174
Ratio FOXP3stroma/ CD4tumor High vs. low	46.6 vs. 81.2	0.012	19.4 vs. 37.8	0.013
Ratio FOXP3stroma/ CD8tumor High vs. low	46.4 vs. 74.3	0.025	23 vs. 37.8	0.042
**Immune cells markers (*N*=84)**				
Tumoral CD8^+^ cells High vs .Low	73.9 vs. 40.4	0.021	56.8 vs. 23	0.026
Stromal FOXP3^+^ cells High vs. low	37.2 vs. 68	0.029	17.4 vs. 46.6	0.101
FOXP3^+^ stroma/ CD4^+^ tumor High vs. low	17.4 vs. 66.9	0.024	32.5 vs. 15.3	0.086
FOXP3^+^ stroma/ CD8^+^ tumor High vs. low	17.4 vs. 68.8	0.011	15.3 vs. 35.9	0.035

NR, not-reached; OS, overall survival; PFS, progression free survival. The *p*-value was calculated using the log-rank test.

**Figure 2:**
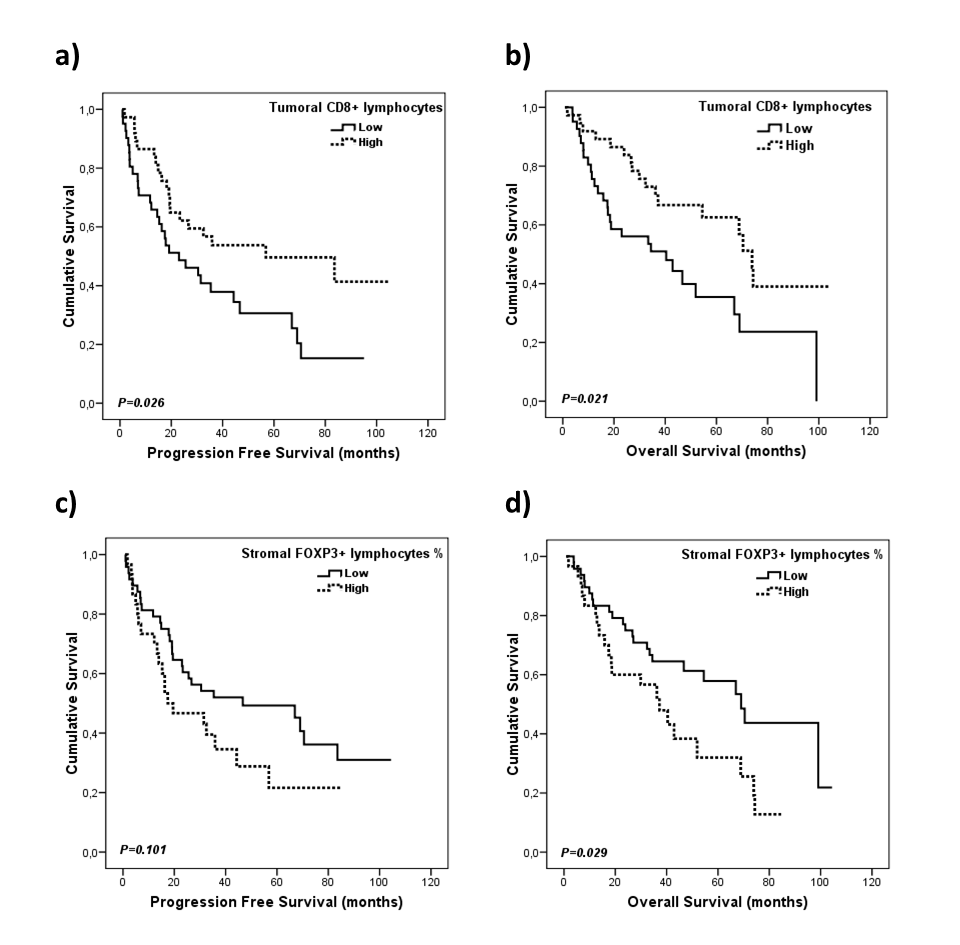
Kaplan-Meier plots for progression free survival and overall survival according to immune cells infiltration **A.**, **B.** Infiltration of CD8^+^ cells in tumor compartment and, **C.**, **D**. infiltration of FOXP3^+^ cells in the stroma compartment. Densities were dichotomized as high and low infiltration according to the median of positive lymphocytes..

As for the immune cells markers detected by IHC, the presence of CD8+ cells in the tumor compartment, according to the median of positive cells infiltrating the tumor, was significantly associated with better PFS (56.8 *vs*. 23 months, *p* = 0.026) and OS (73.9 *vs*. 40.4 months, *p* = 0.021; Figure 2A-2B), whereas patients with a higher percentage of FOXP3+ cells in the stromal compartment (more that 10% of positive lymphocytes) had shorter OS (37.2 *vs*. 68 months, *p* = 0.029; Figure 2C-2D; Table 2).

### Prognostic value of combined biomarkers

**Figure 3:**
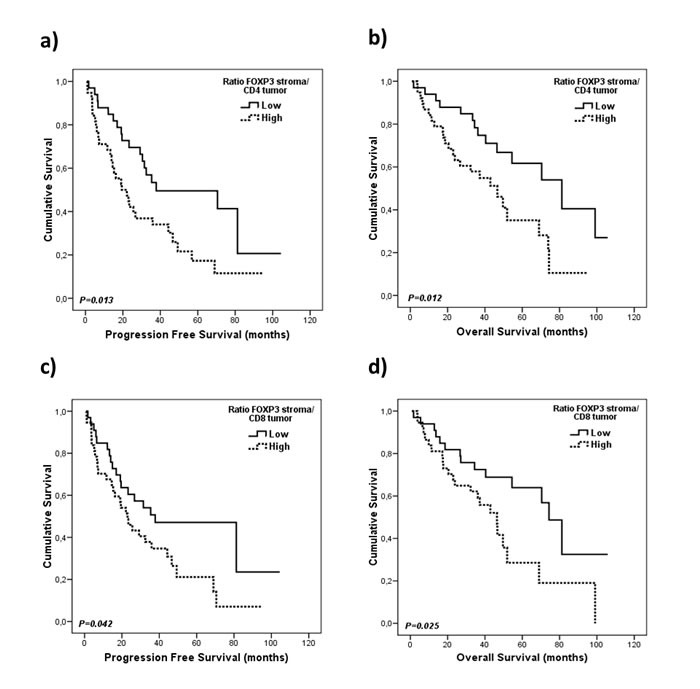
Kaplan-Meier plots for progression free survival and overall survival according to the ratios for conventional and regulatory gene expression markers **A.**, **B.** FOXP3 stroma/ CD4 tumor expression ratio and, **C.**, **D.** FOXP3 stroma/ CD8 tumor expression ratio. Gene expression levels were dichotomized according to the median.

According to these results, and taking into account the biological interactions between the immune cells that express these markers, we decided to further study the prognostic value of the combination of conventional T cells (CD4 and CD8) and Tregs (FOXP3). First, we calculated new variables based on the ratio of these markers and used the median to dichotomized them as “low” or “high”. From the different combinations that were tested we observed that patients with a high FOXP3 stroma/ CD4 tumor expression ratio have reduced PFS (19.4 *vs*. 37.8 months, *p* = 0.013) and OS (46.6 *vs*. 81.2 months, *p* = 0.012; Figure 3A-3B; Table 2). As for the FOXP3 stroma/ CD8 tumor expression ratio, patients with high levels also presented worse PFS (23 *vs*. 37.8 months, *p* = 0.042) and OS (46.4 *vs*. 74.3 months, *p* = 0.025; Figure 3C-3D; Table 2).

**Figure 4:**
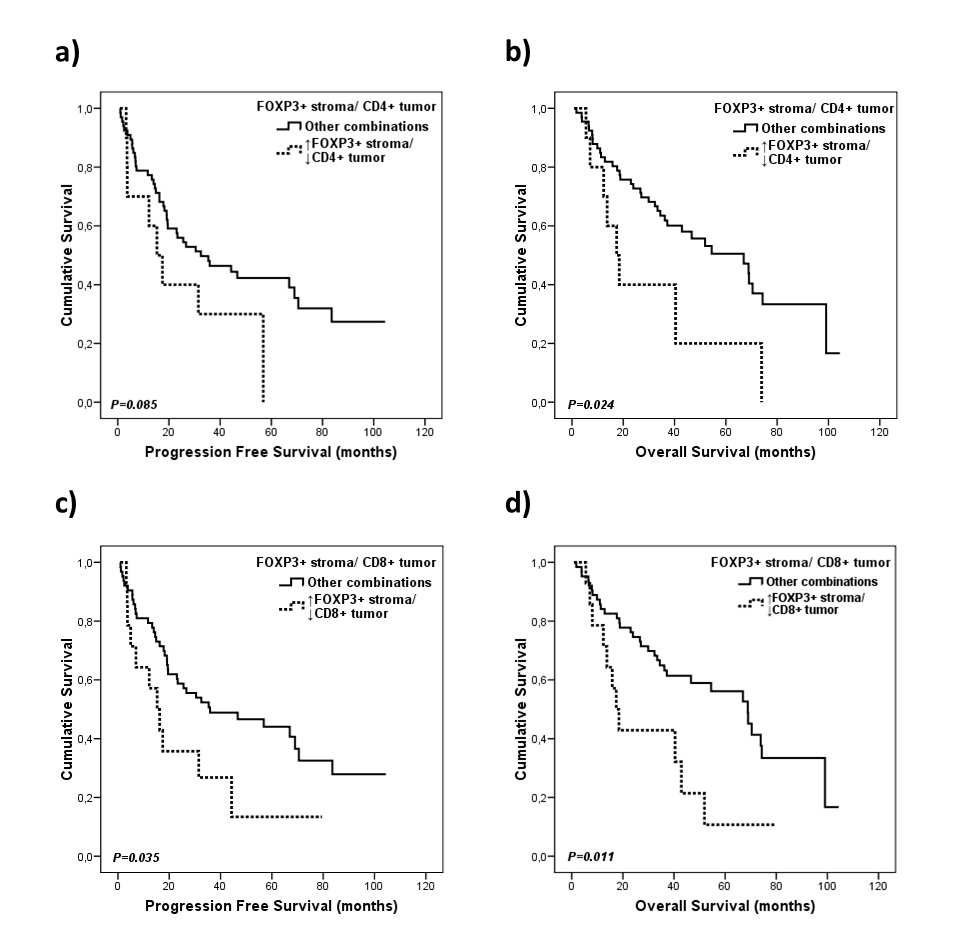
Kaplan-Meier plots for progression free survival and overall survival according to the FOXP3 cells proportion to conventional T cells markers **A.**, **B.** Combination of FOXP3^+^ cells in the stroma/CD4^+^ cells in the tumor, and **C.**, **D.** combination of FOXP3^+^ cells in the stroma/CD8^+^ cells in the tumor.

To validate these results, available tissue samples from 84 of the122 patients were also assessed by IHC. Univariate survival analyses showed that patients with high FOXP3+ cells infiltration (more than 10% of positive lymphocytes) in the stroma and low CD4+ cells infiltration in the tumor (according to the median of positive cells infiltrating in the tumor) presented significantly worse OS (17.4 *vs*. 66.9 months, *p* = 0.024; Figure 4A-4B; Table 2). Moreover, patients with high infiltration of FOXP3+ cells in the stroma and low infiltration of CD8+ in the tumor also presented inferior PFS (15.3 *vs*. 35.9 months, *p* = 0.035) and OS (17.4 *vs*. 68.8 months, *p* = 0.011; Figure 4C-4D; Table 2).

### Multivariate analysis

To determine whether any of the analyzed variables had independent prognostic impact, a multivariate analysis was performed, where significant biomarker and clinicopathological variables from the univariate analyses were entered in the analysis. Multivariate models for gene expression markers and for the immune cell markers assessed by IHC were carried out separately due to the different number of patients analyzed, and results are summarized in Table 3. As for the multivariate model including gene expression markers, PFS was adjusted for tumor size, which was significanty associated with PFS in the univariate analysis. The expression of CD8 in the tumor compartment was found to be an independent beneficial prognostic factor for both PFS (*p* < 0.001) and OS (*p* < 0.001).

**Table 3:** Results from the multivariate Cox regression model for OS and PFS

	OS			PFS		
Variable	HR	95% CI	*p*-value	HR	95% CI	*p*-value
**Gene expression markers (*N*=122)**						
Tumoral CD8 expression High vs. low	0.169	0.064-0.447	<0.001	0.247	0.120- 0.507	<0.001
**Immune cells markers (*N*=84)**						
Tumoral CD8^+^ cells High vs. Low	0.386	0.175-0.850	0.018	0.305	0.137-0.680	0.004
Stromal FOXP3^+^ cells High vs. Low	2.203	1.109-4.379	0.024	-	-	-

CI, confidence interval; HR, hazard ratio; OS, overall survival; PFS, progression free survival.

A second multivariate model including immune cells markers determined by IHC was performed as well. In this model lymph node involvement and PS were included for both OS and PFS due to their significant correlation with survival observed in the univariate analysis. Here, results indicated that the presence of CD8+ cells in the tumor compartment and FOXP3+ cells in the stromal compartment, were independent prognostic biomarkers for OS (*p* = 0.018 and *p* = 0.024, respectively), and CD8+ cell infiltration in the tumor compartment was an independent prognostic factor also for PFS (*p* = 0.004).

## DISCUSSION

Over the past decade, the field of tumor immunology has been increasingly investigated. Today, it is generally accepted that the immune system plays a pivotal role in cancer, and provides prognostic and predictive markers. In our study, we have found that gene expression levels of CD8 in both tumor and tumor-near stroma compartments, and of CD4 only in the tumor compartment, were correlated with better outcomes. Furthermore, when the infiltration of cells positive for these markers was assessed by IHC, only the presence of CD8+ cells in the tumor compartment was correlated with survival.

Although TILs have been widely assessed in the tumor microenvironment by IHC, little has been published regarding the expression of TIL-associated genes at the mRNA level. The latter approach is challenging because while it is highly feasible to distinguish between tumor *versus* stromal compartments by microscopy (IHC), this is more demanding when using RTqPCR, despite carefully performed tissue microdissections. On the other hand, the RTqPCR methods are more sensitive and objective, whereas IHC data are semiquantitative with the inconvenience that it may infer.

Among TILs, CD8+ cells form the effector arm of adaptive immunity with cytotoxic activity and are considered to have tumor preventive effects. We have observed that the presence of CD8+ cells in the tumor compartment, detected either by IHC or by RTqPCR, is highly associated with a beneficial prognosis. Accordingly, Ruffini *et al.* observed that CD8+ cells were associated with prolonged survival in 1290 stage I-IIIA NSCLC patients, but that an independent prognostic impact was associated with squamous cell carcinomas only [30]. At about the same time Al-Shibli *et al.* observed that high CD8+ lymphocyte infiltration, in both tumor and stromal areas, was associated with better survival in 335 resected stage I-IIIA NSCLC patients [10]. Recently, it has been demonstrated that the density of stromal CD8+ cells, due to its significant independent prognostic impact, might be a good candidate marker for establishing a TNM-Immunoscore in resected NSCLC [11].

In contrast, the presence of CD4+ cells in different types of cancer [31-33] has yielded contradictory results regarding prognosis. In lung tumors, the presence of stromal CD4+ lymphocytes has, in general, been correlated with improved survival [10, 34, 35]. Our results obtained by IHC are consistent with previous NSCLC studies. In a 50-gene signature study of stage I/II NSCLC tumors the presence of CD4+ cells did not correlate with survival [36]. This discrepancy can be explained by the fact that CD4+ TILs form a heterogeneous population of cells with different phenotypes and even with opposing actions in the tumor microenvironment [9].

In our study, FOXP3+ provided opposite results according to compartment analyzed and methodology. While FOXP3 gene expression levels in the tumor were significantly associated with better outcomes, the presence of FOXP3+ cells in the stroma had the opposite effect. In previous studies with different types of cancer, the presence of FOXP3+ cells has been associated with both poor [37, 38] and improved OS [39-41], although mRNA expression levels were not assessed in any of these studies. A possible explanation for this observation is the extreme heterogeneity of resected carcinomas, the mixed patterns of adenocarcinomas [42], and the fact that we could not select areas representative of all the major tissue patterns present. Additionally, cancer cells positive for FOXP3 have been detected in several distinct types of cancer [37,43] that could attenuate the unfavorable prognostic influence of the tumor-infiltrating Tregs detected by IHC. In contrast, the presence of FOXP3+ cells or Tregs in the stroma was correlated with worse survival. Shimizu *et al.* demonstrated that patients with NSCLC (stages I-IIIB) containing three or more infiltrating Treg cells/10 HPFs in the tumor tissue had significantly worse recurrence-free survival (RFS), and among patients with node-negative NSCLC, Tregs were an independent poor prognostic factor [38]. An increased Treg count was also found to be associated with worse OS and RFS in another study with I-IIIA stage NSCLC [37]. Furthermore, a high density of stromal FOXP3+ cells was associated with a shorter recurrence-free probability [44]. Another possible explanation for this results may come from the heterogeneous phenotypes described for FOXP3+ regulatory T lymphocytes [45], their epigenetic regulation [46] or the presence of exhausted phenotypes [47]. Our results indicate that there is a great variability of each particular subpopulation between patients and this aspect could affect to the designation of a potential biomarker. This variability can be explained because immune cells are more prone to be located in the stroma than within the tumor compartment. As for the difference between the particular subpopulations, it was expected to obtained similar data for CD4 and CD8 positive cells, but not in the FOXP3 positive cells case. This is due to the fact that FOXP3 cells or regulatory T cells are less common than conventional T cell. All these observations emphasize the importance of assessing the location and type of immune cells within the tumor microenvironment.

Our data shows that patients with high expression levels of LAG3 in the tumor compartment presented a better outcome. LAG3 functions as an immune checkpoint and has a role in enhancing the function of Treg cells [48]. To the best of our knowledge, the prognostic value of LAG3 has not previously been assessed in NSCLC or any other tumor type. Another marker that presented a significant association with OS when expressed in the tumor compartment was TGFB1. This marker plays a critical and dual role in the progression of human cancer [49-52] and ithat TGFB1 positive patients showed remarkably longer survival than the others [53].

We also studied the prognostic value of conventional TILs such as CD4+ and CD8+ cells in combination with FOXP3+ cells. In addition to type, density, and location, we have also demonstrated that the relative proportion of pro- and antitumor immune cells should be studied when analyzing TILs. According to our data, the ratio of FOXP3+ *vs* CD4+ or CD8+ cells in both the tumor and stromal compartment had a prognostic value. In this regard, it was observed that patients with stage I NSCLC with a higher proportion of tumor Tregs relative to CD3+ TILs, a pan-T cell marker that does not differentiate among helper and cytotoxic T cells, had a significantly higher risk of recurrence [54]. In a previous study including stage I adenocarcinomas, tumors containing high stromal FOXP3+ and low stromal CD3+ were considered to be more progressive, and FOXP3+ high *versus* stromal CD3+ low was found to be a strong predictor of recurrence [44]. To the best of our knowledge, our study is the first reporting the prognostic value of Tregs *versus* cytotoxic or helper TILs in the tumor or tumor-near stromal compartment.

In the multivariate analysis, CD8 gene expression by means of RTqPCR and infiltrating CD8+ cells evaluated by IHC in the tumor compartment were found to be independent prognostic factors for OS and PFS, probably indicating an ongoing immune response against the tumor [55]. Furthermore, FOXP3+ cells in the tumor-near stroma compartment was an independent negative prognostic factor for OS, emphasizing the importance of assessing the location of immune cells within the tumor microenvironment.

In conclusion, although some papers address the role in CD8 and Foxp3 in lung cancer, the original aspects of this study lies in the fact that the tumor and stroma compartment of the tumor tissue have been investigated separately with a double approach using IHC and RTqPCR. Thus, the presence of CD8+ TILs in the tumor compartment, both at mRNA level and detected by IHC, identified a group of patients with longer survival, suggesting that an activated immune response might have an important role in patient outcome. In contrast, the presence of FOXP3+ cells in the stromal compartment identified a subset of patients with worse survival. This association was also specifically observed when the density of CD8+ and CD4+ in the tumor was low, but not in other cases, suggesting the importance of assessing the relative proportion of conventional and regulatory T cells present in the tumor surroundings. Further investigations and prospective clinical studies will be required to validate these results and their possible therapeutic implications.

## MATERIALS AND METHODS

### Patients and tissue samples

In this retrospective study, 122 patients from *Consorcio Hospital General Universitario de Valencia* with a clinical and histological diagnosis of stage I to IIIA (according to the American Joint Committee on Cancer staging manual) NSCLC and diagnosed between 2004 and 2013, were included. None of the patients received chemo- or radiotherapy prior to surgery. The study was conducted in accordance with the Declaration of Helsinki, and the institutional ethical review board approved the protocol. Reported recommendations for tumor marker prognostic studies (REMARK) criteria were followed throughout the study [56]. Progression-free survival (PFS) was estimated as the time from surgery to recurrence or death from the disease, whereas overall survival (OS) was defined as the time from diagnosis to the date of death or last follow up. Formalin-fixed, paraffin-embedded (FFPE) samples obtained from tumor resections in these patients were used in this study.

### Gene expression analysis

Laser capture microdissection was performed in order to separately collect tumor and tumor-near stroma tissue areas from 122 FFPE tumor specimens. For this purpose, a LMD6500 Leica laser microdissection system (Leica, Wetzlar, Germany) was used with the support of a Hitachi HV-D20 camera (Hitachi Kokusai Electric, Tokyo, Japan). RNA was isolated using a RecoverAll™ Total Nucleic Acid Isolation Kit (Ambion, Austin, TX, USA) following the manufacturer's recommendations. Reverse transcription (RT) reactions were performed from 100 ng of total RNA using random hexanucleotides and a High-Capacity cDNA Reverse Transcription Kit (Applied Biosystems, Foster City, CA, USA) following the manufacturer's instructions. The thermal cycling conditions were as follows: 10 min at 25° C, 120 min at 37° C, and 5 s at 85° C. A TaqMan^®^ PreAmp Master Mix Kit (Applied Biosystems) was used for preamplification to increase the quantity of specific cDNA target. The preamplification conditions were as follows: 10 min at 95° C for enzyme activation, followed by 10 cycles of 15 s at 95° C and, finally, 4 min at 60° C.

RTqPCR was performed to analyze the relative expression of *CD4, CD8, FOXP3, CD25, CD127, CTLA4, LAG3, GITR, TGFB1* and *IL10* genes with assays based on hydrolysis probes using 1 μl of cDNA, a Universal Master Mix, and a TaqMan Gene Expression Assay (Applied Biosystems), in a 5 μl final reaction volume. *GAPDH* and *CDKN1B* were selected as endogenous controls by using the GeNorm algorithm. The thermal cycling parameters were as follows: 2 min at 50°C and 10 min at 95°C followed by 40 cycles of 15 s at 95°C and 1 min at 60°C. For efficiency calculations, we used random-primed qPCR Human Reference cDNA (Clontech, USA). Relative gene expression levels were expressed as the ratio of target gene expression to the geometric mean of the endogenous gene expression by using the Pfaffl formula [57]. We considered a gene to be overexpressed in the tumor or tumor-near stroma compartment when the median of the relative gene expression referred to the human cDNA was higher than 2 and underexpressed when it was less than 0.5. Gene expression levels were dichotomized as “high” and “low” according to the median of each case.

### Immunohistochemistry

In this study, the expression of FOXP3, CD4, and CD8 was evaluated in 84 of the FFPE samples included using a Dako Autostainer Link 48 and the Dako EnVision™ FLEX detection system (Dako, Burlington, Canada). Briefly, sections were dried and loaded into the PT Link instrument where the antigen retrieval/dewaxing process took place. The sections were transferred to the Autostainer Link 48 instrument where the endogenous peroxidase activity was quenched with peroxidase blocking reagent for 10 minutes. Immunostaining was carried out with Dako FLEX Ready to-Use format for CD4 (Clone 4B12, Dako) and CD8 (Clone C8/144B, Dako), and with a primary antibody diluted at 1:300 with Dako Antibody Diluent (Dako) for FOXP3 (Clone 236A/E7, Abcam, Cambridge, MA, USA). After incubation with the primary antibody a detection system chromogen (3,3’-diaminobenzidine, DAB) was used. Finally, the sections were washed, lightly counterstained with hematoxilin, dehydrated, and mounted. Normal human tonsil tissue was used as a positive control for the three antibodies, and negative controls were included by omitting the primary antibody.

### Quantification of immunohistochemical staining

The slides were independently examined by two researchers (SC, JF or MM), all were blinded to cases and data. Before initiating the scoring, controls were reviewed for quality assurance, compartments for scoring were agreed upon and the semiquantitative scale to be used was defined. To evaluate FOXP3 immunostaining, the percentage of nuclear-stained lymphocytes present in the tumor and tumor-near stroma was defined and graded on a scale of 0-3 according to the percentage of positive lymphocyte cells: 0 = no staining, 1 = less than 10% positive, 2 = 10-32% positive, 3 = 33% or more positive. Furthermore, high power fields (HPFs; magnification X400) were analyzed in 10 tumor or stromal areas per sample and the absolute number of FOXP3+ lymphocytes was determined and then averaged. CD4+ and CD8+ lymphocytes were also counted in 10 HPFs for both tumor and tumor-near stroma areas, and were then averaged. We used 10% positive lymphocytes as a low-high cutoff value for FOXP3+ cell dichotomization. High power fields (HPFs; magnification X400) were analyzed in 10 tumor or stromal areas per sample and the absolute number of CD4+ and CD8+ cells was determined, and the median was obtained in each case. The median of positive cells was used as a cutoff in both cases. Examples of IHC images representing different infiltration scores in tumor and tumor-near stroma locations are shown in Figure 1.

### Statistical analysis

Continuous variables were compared by non-parametric Mann-Whitney U and Kruskall-Wallis tests. A Spearman rank test was used to test for correlations between continuous variables. Correlated variables were not included in the same multivariate model and the association between dichotomized variables was evaluated by the Chi-square test. Survival analysis was performed using a univariate Kaplan-Meier (log-rank) test method with clinicopathological variables, dichotomized gene expression marker levels, and density of immune cell infiltration levels. Finally, to assess the independent value of the tested biomarkers, a Cox proportional hazard model for multivariate analyses was used. All significant variables from the univariate analyses were entered into the multivariate analyses in a forward stepwise Cox regression analysis. A probability of 95% (*P* < 0.05) was considered statistically significant for all analyses. The statistical analyses were done using the Statistical Package for the Social Sciences (SPSS, Chicago, IL, USA) version 15.0.

## SUPPLEMENTARY MATERIAL FIGURE


