# Supplementary material for: Analysis of the immune microenvironment in resected non-small cell lung cancer: the prognostic value of different T lymphocyte markers
Source: Oncotarget. 2016 Jul 24;7(33):52849–61. doi: 10.18632/oncotarget.10811 (PMC5288153; doi:10.18632/oncotarget.10811)
Supplement: Supplementary file 1 [file oncotarget-07-52849-s001.pdf]

Analysis of the immune microenvironment in resected non-small cell lung cancer: the prognostic value of different T lymphocyte markers

Supplementary Material

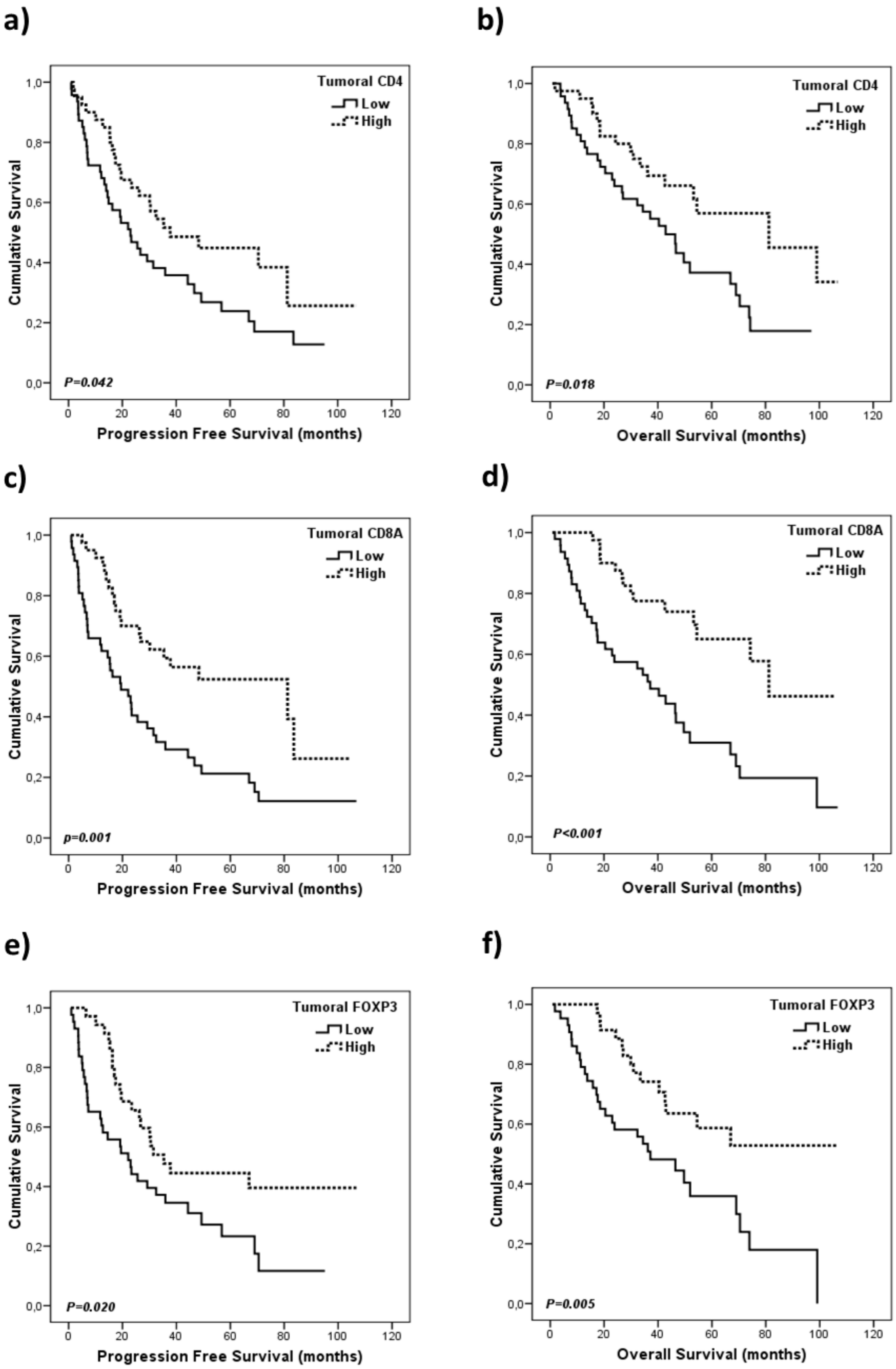

Supplementary Figure 1. Kaplan-Meier plots for progression free survival and overall survival according gene expression markers. (A, B) Tumoral CD4, (C, D) tumoral CD8 and, (E, F) tumoral FOXP3. Gene expression levels were dichotomized as high and low according to the median.
